# Visualization of distribution in the vitreous cavity via eye drops using ultra-heavily T2-weighted sequences in MRI: a preliminary study with enucleated pig eyes

**DOI:** 10.1007/s12194-024-00826-6

**Published:** 2024-07-18

**Authors:** Yutaka Kato, Kenya Yuki, Koji Nishiguchi, Shinji Naganawa

**Affiliations:** 1 Department of Radiological Technology, Nagoya University Hospital, 65 Tsurumai-Cho, Shouwa-Ku, Nagoya, Aichi 466-8560 Japan; 2 Department of Ophthalmology, Nagoya University Graduate School of Medicine, 65 Tsurumai-Cho, Shouwa-Ku, Nagoya, Aichi 466-8560 Japan; 3 Department of Radiology, Nagoya University Graduate School of Medicine, 65 Tsurumai-Cho, Shouwa-Ku, Nagoya, Aichi 466-8560 Japan

**Keywords:** Eye drop, Intraocular distribution, Magnetic resonance imaging, ^17^O, Gadolinium

## Abstract

We investigated whether magnetic resonance imaging can visualize the distribution in the vitreous cavity via eye drops of ophthalmic solutions, gadolinium-based contrast agent, and ^17^O-water, and to clarify the usefulness of ultra-heavily T2-weighted sequences in the research of intraocular distribution. Five different solutions (V-ROHTO, TRAVATANZ, gadobutrol, H_2_^17^O, and saline) were administered to excised pig eye specimens. The samples were scanned using T1 mapping, T2 mapping, 3D T2-weighted (echo times (TE): 500, 3200, and 4500 ms), a half-Fourier single-shot turbo-spin echo sequence (HASTE; TE: 440 and 3000 ms), and 3D-real inversion-recovery before eye drops administration. Subsequently, we used a plastic dropper to drop a 0.5 mL solution each, and images were obtained up to 26 h later. Temporal changes in the T1 and T2 values of the anterior chamber and vitreous cavity were compared. The other sequences were evaluated by determining temporal signal changes as signal intensity ratio (SIR) compared to “No drop.” The T1 and T2 values of samples treated with gadobutrol and H_2_^17^O decreased over time. The SIR of samples treated with gadobutrol and H_2_^17^O showed remarkable changes in the 3D T2-weighted images, whereas no remarkable temporal changes were observed in the other solutions. Longer TEs resulted in remarkable changes. We demonstrated that visualization of distribution in the vitreous cavity via eye drops could be achieved with excised pig eyes using gadobutrol and H_2_^17^O, but not with ophthalmic solutions. Ultra-heavily T2-weighted sequences may be promising for the early and highly sensitive visualization of the intraocular distribution of eye drops.

## Introduction

Penetration of topical eye drops into the anterior chamber and vitreous cavity of the eye is an important issue in ocular pharmacology [1]. It is anticipated that a visualization of aqueous humor flow will contribute to elucidating the mechanisms of ocular diseases such as glaucoma. Previous studies using radioactive isotopes have reported the intraocular penetration of topically instilled eye drops [2, 3], with those containing isotopically labeled 1% [14C] nipradilol, a glaucoma drug, being detectable in the anterior chamber and posterior retina of the eyes of rabbits [2] and monkeys [3]. However, these reports using radioactive isotopes are difficult to use in human subjects, and experiments are difficult to perform. In contrast, magnetic resonance imaging (MRI) is safe for human subjects and can potentially be used to observe the intraocular distribution of drugs. Several MRI studies in rabbits and humans have reported the distribution of the anterior chamber via eye drops using deuterium oxide (D_2_O) [4], gadolinium-based contrast agents (Gd) [5], and ^17^O-labeled water (^17^O) [6, 7]. However, drug distribution in the vitreous cavity has not been well observed in previous studies [5–7] and remains challenging.

Sequence optimization is a critical factor in visualization. ^17^O can be captured as a signal change caused by T2 shortening [8]. Therefore, previous studies have been performed using heavily T2-weighted images (e.g., echo times (TE) of 360 ms [6] and 444 ms [7]). Recently, ultra-heavily T2-weighted imaging (UH-T2W) with a TE of 3200 ms, which is more sensitive to signal changes caused by ^17^O administration, was proposed for an inner ear study [9]. Furthermore, 3D inversion-recovery with real reconstruction imaging (3D-real IR), which is sensitive to T1 changes, has been used in studies on endolymphatic hydrops [10], gadolinium leakage [11, 12], and glymphatic systems [13]. We hypothesized that these high-sensitivity sequences would provide visualization of the distribution in the vitreous cavity via eye drops.

The selection of a topical eye drop solution is another important factor in the visualization of intraocular distribution. An eye drop of Gd is off-label, and the use of ^17^O is costly. It would be ideal if the clinical ophthalmic solutions could be observed using MRI. Ophthalmic solutions have different components depending on type, implying that the T1 and T2 values of the solutions would differ by type. However, to the best of our knowledge, no such study has been conducted.

The purpose of this study was to investigate whether MRI can visualize the distribution in the vitreous cavity via eye drops of ophthalmic solutions, in addition to the Gd and ^17^O used in previous studies, and to clarify the usefulness of UH-T2W in research on intraocular water penetration and distribution.

## Materials and methods

### Selection of ocular drugs

We measured the T1 and T2 values of several ophthalmic solutions as a preliminary step (the types and values are listed in Table 1) and then decided to use solutions with short T2 values. This is because the measured ophthalmic solutions had similar T1 values but widely different T2 values, and we expected that a solution with a short T2 value would decrease the signal of the vitreous cavity with a relatively long T2 value. For this experiment, commercially available ophthalmic solutions (V-ROHTO premium; ROHTO Pharmaceutical Co., Ltd., Osaka, Japan) and therapeutic ophthalmic solutions (Travoprost; TRAVATANZ; Alcon Japan Ltd.) were prepared. Gd (gadobutrol; Gadovist; Bayer Pharma, Osaka, Japan), ^17^O (10 mol% H_2_^17^O; Taiyo Nippon Sanso, Tokyo, Japan), and saline were also prepared. The T1 and T2 values for each solution are summarized in Table 2; the values of Gd were too small to measure. All T1 and T2 values were obtained using the inversion-recovery and multi-spin-echo methods, respectively.

**Table 1:** T1 and T2 values of the ophthalmic solutions in the preliminary step

	AIPHAGAN	GLANATEC	GLA-ALPHA	Carteolol	LUMIGAN	TAPROS	TRAVATANZ	Xalatan	V-ROHTO
T1 value (ms)	2730	2646	2678	2480	2782	2672	2668	2715	2612
T2 value (ms)	798	1240	1826	422	1854	690	295	2140	391

**Table 2:** T1 and T2 values for each solution used in this study

	Gadobutrol	10 mol% H_2_^17^O	V-ROHTO	TRAVATANZ	Saline
T1 value (ms)	Not measurable	2500	2612	2668	2800
T2 value (ms)	Not measurable	50	391	295	2294

### Sample: enucleated pig eyes

**Fig. 1:**
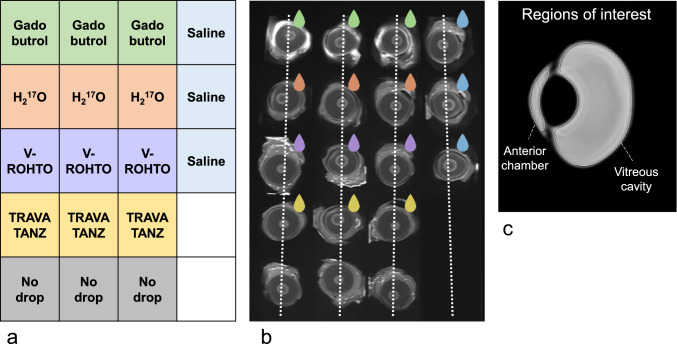
Layout of topical eye drop solutions and imaging plane settings for 2D sequences. Five different solutions were dropped for three eyes each (**a**). Three eyes were prepared without drops. The 2D sequence was scanned with one slice in each row (**b**, *dotted line*). Regions of interest were drawn in the anterior chamber and vitreous cavity by the autocontouring function in the MR console (**c**)

Eighteen freshly enucleated porcine eyes were prepared and placed in plastic grid boxes. Five different solutions can be administered to three eyes each, and the “No drop” group (three eyes) can be prepared as a control (Fig. 1). The samples were left for several hours before scanning to keep them at room temperature.

### MRI acquisition

All scans were performed using a 3T MRI scanner (Skyra; Siemens Healthcare, Erlangen, Germany) with a 20-channel head-neck receiver coil. The samples were scanned using T1 mapping, T2 mapping, 3D-T2W (TE: 500, 3200, and 4500 ms), half-Fourier single-shot turbo-spin echo sequence (HASTE; TE: 440 and 3000 ms), and 3D-real IR before topical eye drop administration. The 3D-real IR was additionally obtained as an optional imaging to detect slight T1 changes for Gd-administration. The detailed scan parameters are presented in Table 3. The 3D sequences (T1 mapping, 3D-T2W, and 3D-real IR) were scanned in the sagittal plane to cover all samples. The 2D sequences (T2 mapping, HASTE) were scanned in one slice in each row to obtain a sagittal midline image of each eye (Fig. 1b, dotted line).

**Table 3:** Detailed imaging parameters for all sequences

	T1 mapping	T2 mapping		3D-T2W		3D-real IR	HASTE	
Type	3D GRE	2D multi-SE	3D TSE	3D TSE	3D TSE	3D IR (TI, 2250 ms)	2D ss-TSE	2D ss-TSE
Repetition time (ms)	5.0	5000	2500	16,000	16,000	15,000	10,000	10,000
Echo time (ms)	1.94	32 echo (20–640)	500	3200	4500	537	440	3000
Flip angle (°)	2 and 10	180	110	140	140	110	120	120
Spatial resolution (mm)	0.98 × 0.98	0.99 × 0.87	0.56 × 0.56	0.56 × 0.56	0.56 × 0.56	0.80 × 0.56	0.56 × 0.56	0.56 × 0.56
Slice thickness (mm)	3.0	4.0	1.0	1.0	1.0	1.0	4.0	4.0
Bandwidth (Hz/Pixel)	260	299	429	429	199	429	399	192
Number of slices	60	4	208	208	208	160	4	4
Number of averages	1.0	1.0	1.4	2.0	2.0	1.0	1.0	1.0
Parallel imaging	No	GRAPPA of 3	GRAPPA of 3	GRAPPA of 3	GRAPPA of 3	GRAPPA of 3	No	No
Turbo factor (data duration)	-	-	140 (863)	599 (4313)	599 (5523)	280 (1215)	448	672
Acquisition time (min:s)	1:09	5:55	4:00	8:48	8:48	5:30	0:42	0:42

*3D* three-dimensional, *T2W* T2-weighted, *IR* inversion recovery, *HASTE* half-Fourier single-shot turbo-spin echo, *GRE* gradient echo, *2D* two-dimensional, *SE* spin echo, *TSE* turbo spin echo, *TI* inversion time, *ss-TSE* single-shot turbo spin echo, *GRAPPA* generalized autocalibrating partial parallel acquisition

Following pre-scanning, we used a plastic dropper to drop a 0.5 mL solution vertically toward the cornea of the pig eye. Subsequently, images were obtained with all sequences in the following timetable: 0.5, 1, 2, 3, 4, 8, 12, and 26 h. Note that the entire sequence takes 30 min in total, and there is a time difference between each sequence and not an exact time point; for example, 0.5 h means 20–50 min after administration. The imaging order was as follows: T1 mapping, T2 mapping, 3D-T2W with a TE of 500 ms, 3D-T2W with a TE of 3200 ms, 3D-T2W with a TE of 4500 ms, 3D-real IR, HASTE with a TE of 440 ms, and HASTE with a TE of 3000 ms.

### Image analysis

Regions of interest (ROIs) were drawn in the anterior chamber and vitreous cavity from a sagittal slice of each eye using the autocontouring function in the MR console (Fig. 1c). The T1 and T2 values were calculated as the average of three eyes from T1 and T2 mapping, respectively. Temporal changes in T1 and T2 values of the anterior chamber and vitreous cavity treated with each solution were compared. The other sequences were measured using signal intensities (SIs) obtained from ROIs of the anterior chamber and vitreous cavity and evaluated using signal intensity ratio (SIR), which is calculated by dividing SI of each sample by the average of three SI for “No drop.” Temporal changes in the SIRs of the anterior chamber and vitreous cavity after administration of each solution were compared. Note that SIR measurements were performed only for T2-weighted sequences, because they were for comparison between different TEs and sequences.

## Results

**Fig. 2:**
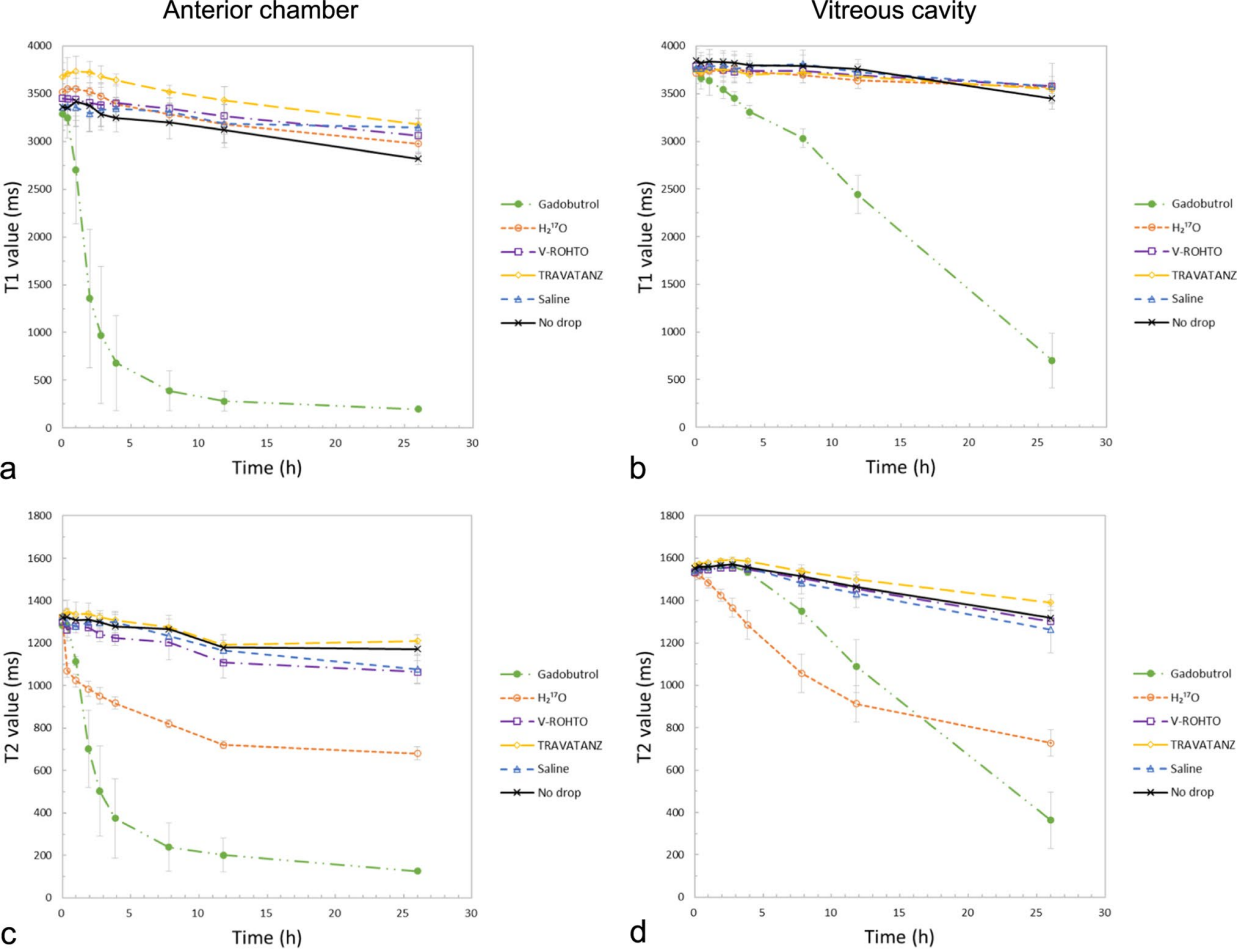
Temporal changes in T1 (**a**, **b**) and T2 (**c**, **d**) values in the anterior chamber and vitreous cavity with the administration of each topical eye drop solution

Figure 2 shows the temporal changes in T1 and T2 values in the anterior chamber and vitreous cavity after administration of each solution. The T1 value of the samples administered with Gd decreased rapidly in the anterior chamber and gradually in the vitreous cavity. Other solutions showed similar changes to “No drop,” i.e., no significant findings (Fig. 2a, b). T2 values of samples decreased for Gd and ^17^O, whereas the others showed similar changes to “No drop”; ^17^O decreased first, then Gd decreased drastically (Fig. 2c, d).

**Fig. 3:**
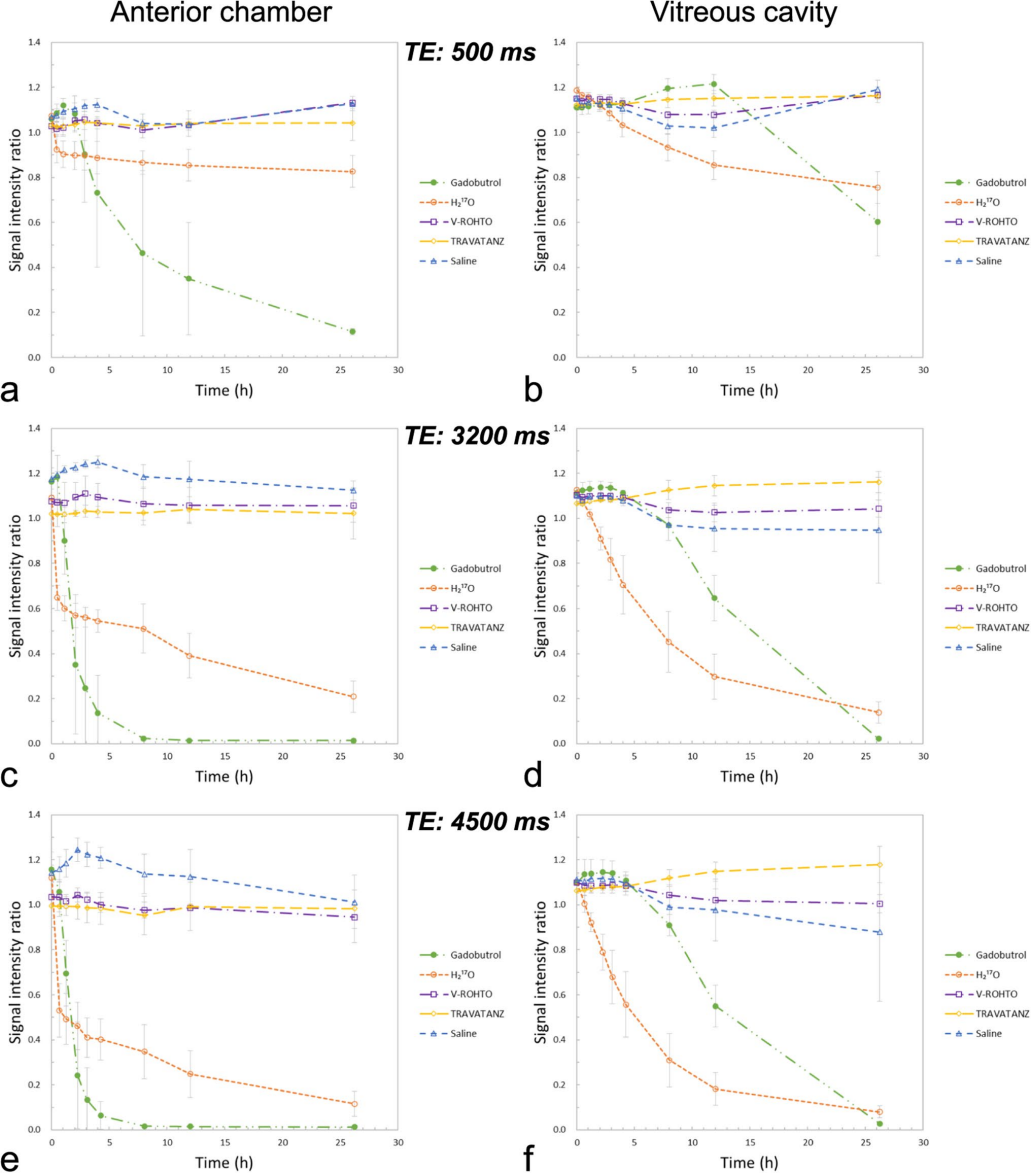
Temporal changes in signal intensity ratio (SIR) using 3D T2-weighted sequences in the anterior chamber and vitreous cavity with the administration of each topical eye drop solution. Echo time (TE) of 500 ms (**a**, **b**), 3200 ms (**c**, **d**), and 4500 ms (**e**, **f**). The SIRs with gadobutrol and H_2_^17^O showed temporal changes, whereas the others showed no obvious changes. The application of longer TEs resulted in a remarkable SIR change

**Fig. 4:**
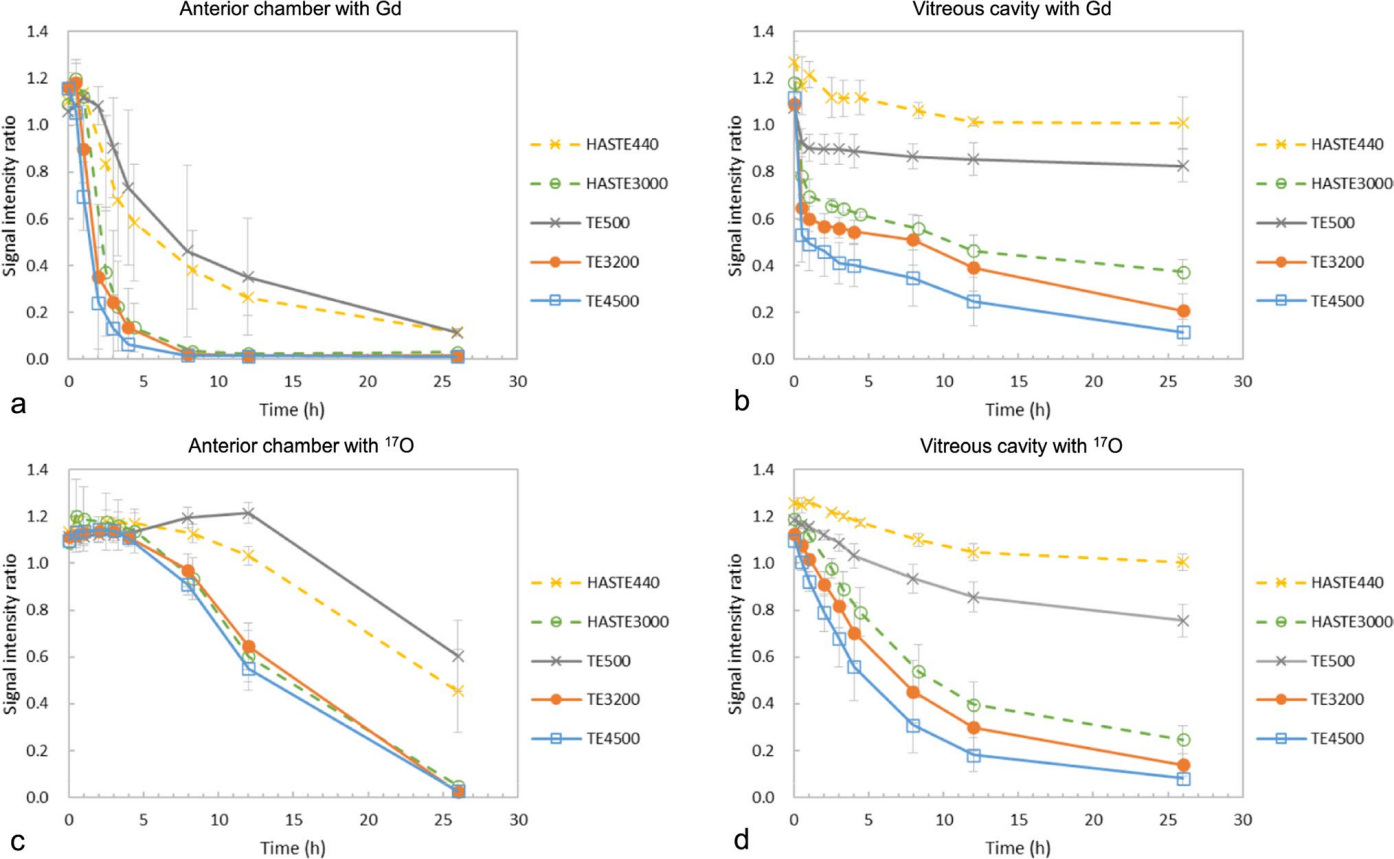
Sequence comparison of signal intensity ratio (SIR) in the anterior chamber and vitreous cavity with the administration of gadobutrol (Gd) and H_2_^17^O (^17^O). The sequence is as follows: 3D T2-weighted with echo time (TE) of 500, 3200, and 4500 ms, HASTE with TE of 440 and 3000 ms. The SIRs for TE500 and HASTE440 showed similar changes; even the HASTE sequence provided great signal changes by applying a super-long TE

Figure 3 shows the temporal signal changes in the 3D-T2W sequences. Similar to the T2 value changes, the SIR of samples administered with Gd and ^17^O showed remarkable changes, whereas no remarkable temporal changes were observed in the other solutions. Figure 4 shows a comparison of the sequences of SIR in 3D-T2W and HASTE for Gd and ^17^O, with the data obtained for 3D-T2W being the same as that shown in Fig. 3. Longer TEs showed more remarkable changes, indicating that both sequences can be highly sensitive to signal changes by applying a super-long TE.

**Fig. 5:**
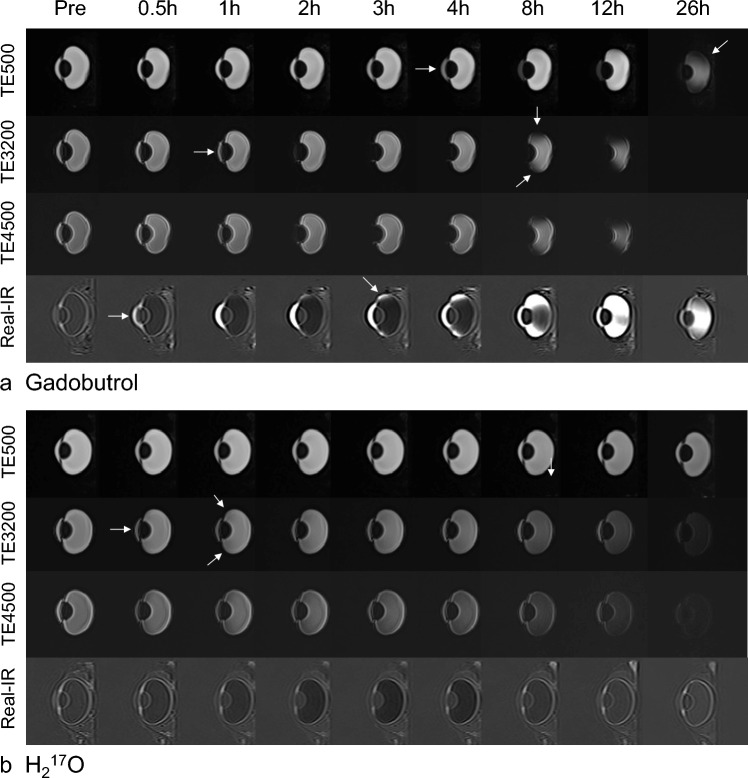
Representative images. Enucleated pig eyes imaging with the administration of gadobutrol (**a**) and H_2_^17^O (**b**) in 3D T2-weighted and 3D-real IR sequences. Arrows indicate the timing of the signal change

Figure 5 shows representative images (presented only for the samples treated with Gd and ^17^O). After Gd administration, the TE500 image showed a visual signal decrease in the anterior chamber at 4 h and a strong signal decrease in the vitreous cavity at 26 h; the TE3200 image showed a visual signal decrease in the anterior chamber at 1 h and a decrease in the vitreous peripheral signal at 8 h. After ^17^O administration, the signal decrease in the anterior chamber was observed on the graph for TE500 (Fig. 3a), but it was visually obscure, and there might be a slight signal decrease visually in the vitreous cavity after 12 h. The TE3200 image showed a visual signal decrease in the anterior chamber at 0.5 h, and a gradual signal decrease in the vitreous cavity from the periphery at 1 h. In both solutions with TE4500, the tendency for signal decrease was more remarkable compared to TE3200. In 3D-real IR images, only the Gd sample showed a strong signal increase. Signal increases in the anterior chamber were observed at 0.5 h, and their distribution in the vitreous cavity gradually increased at 2 h and later.

## Discussion

The present study demonstrated clear penetration and distribution of eye drops into the vitreous cavity using a novel approach with super-long TE (i.e., UH-T2W) and 3D-real IR sequences. To the best of our knowledge, this is the first study to clearly detect the penetration of eye drugs into the vitreous cavity using standard clinical MRI. In enucleated pig eyes with topically administered Gd and ^17^O, signal changes could be observed visually in the vitreous cavity as well as in the anterior chamber. In contrast, no signal changes were observed visually with clinical ophthalmic solutions and saline; there were similar temporal changes in T1 and T2 values compared to those of the “No drop.”

Previous studies on the topical administration (eye drops) of Gd or ^17^O have observed signal changes only in the anterior chamber and not in the vitreous cavity [5–7]. The present study provided a visualization of drug penetration and distribution to the vitreous cavity by imaging over a longer time than previous studies and using high-sensitivity sequences. Obata et al. used D_2_O to visualize water distribution in the vitreous in rabbits [4]. However, D_2_O imaging requires ^2^H-MRI, and most clinical devices are only compatible with ^1^H; thus, its versatility may be problematic. In this study, the distribution in the vitreous was visualized via eye drop with a ^1^H-MRI. These findings will greatly advance future studies on intraocular aqueous flow dynamics.

We attempted, for the first time, to examine intraocular distribution by administering clinical ophthalmic solutions that had not been examined in previous studies. The use of UH-T2W was expected to allow visualization of the intraocular distribution because the T2 values of the selected solutions from the preliminary tests were sufficiently short (e.g., 295 ms for TRAVATANZ) compared to that of the vitreous cavity (approximately 1500 ms before administration). Unfortunately, the distribution could not be visualized using ophthalmic solutions. The T2 value of the original ^17^O solution was 50 ms, and that of Gd was not measurable (quite short), which would have allowed visualization of the intraocular distributions because of the overwhelmingly short T2 value. Applying an even longer TE than that used for UH-T2W, visualization might be feasible when using clinical ophthalmic solutions. Nevertheless, given that signal changes even in the anterior chamber were not observed in the present study, ophthalmic solutions are considered insufficiently effective with respect to altering the T2 values for the vitreous cavity, compared to Gd and ^17^O. For other viewpoints, there may be less penetration of ophthalmic solution into the vitreous cavity. A previous study demonstrated that azone, a commonly used penetration enhancer, can increase the signal in the anterior chamber [5]. A combination of ophthalmic solutions and penetration enhancers or viscosity-increasing agents may allow visualization of distributions in the vitreous cavity.

**Fig. 6:**
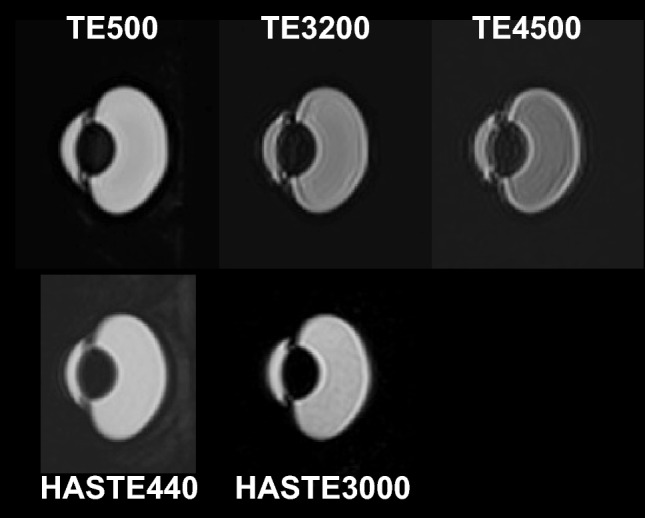
Sample images with 3D T2-weighted and HASTE sequences before topical eye drop administration. Ultra-heavily T2-weighted sequences (TE3200 and TE4500) had blurring artifacts, whereas the HASTE sequence with super long TE (HASTE3000) provided artifact-free images

In this study, 3D-real IR for T1 changes and 3D-T2W with super-long TE for T2 changes were found to be useful for visualizing intraocular distribution. Both sequences are 3D acquisitions, which facilitate the evaluation of detailed structures, such as distribution pathways to the vitreous. However, 3D sequences require long acquisition times, and can cause motion artifacts attributable to eye movements or blinking during scanning, which will inevitably pose problems for future human studies. To address these concerns, Tomiyasu et al. employed a single-shot sequences (i.e., HASTE) in their study on humans [7]. We therefore propose a new approach based on the application of a super-long TE using the HASTE technique, which could provide high sensitivity to T2 changes imaging, comparable to that obtained using 3D-T2W with a TE of 3200 ms (Fig. 4). A further concern is the long data duration of the 3D-T2W, which may cause blurring artifacts. Sample images obtained using 3D-T2W and HASTE sequences are shown in Fig. 6. The 3D-T2W with a TE of 3200 and 4500 ms shows more pronounced blurring artifacts than images obtained using a TE of 500 ms. In contrast, the HASTE sequence provides images without blurring artifacts, even with a TE of 3000 ms. Moreover, images can be obtained within a few seconds per slice. Thus, although it is a 2D acquisition, it can provide motion-robust and high temporal resolution. With the application of UH-T2W, HASTE would be appropriate for observation of aqueous flow dynamics with high temporal resolution in the early phase, and 3D imaging would be appropriate for a detailed anatomical interpretation of intraocular distribution.

Furthermore, the use of high-sensitivity sequences suggests that drop volume can be reduced. Our study showed visualization of intraocular distribution with smaller volumes than in a previous study (0.5 mL in our study, 0.92–1.37 mL in a previous study [7]), but still too large. Future studies are needed to investigate whether signal changes can be observed with a normal dosage (i.e., 1 drop = 0.05 mL) in humans. In addition, several previous studies on Gd eye drop dacryocystography in humans have administered it at a 1:100 dilution [14, 15]. In the present study, the original solution was administered, which may have resulted in significant changes in the signal. We should determine the appropriate dilution rate while preventing adverse effects for living human study.

We also established that there are differences between Gd and ^17^O with respect to the speed and pattern with which these drugs penetrate the vitreous cavity. Compared with Gd, we found that ^17^O administered via eye drops was characterized be a more rapid distribution, which is consistent with the findings of a previous study on glymphatic water transport in the rat brain in vivo [16]. In addition, the penetration of Gd into the vitreous cavity may be initiated from the peripheral through the sclera, whereas in contrast, ^17^O appears to be distributed from the anterior chamber side into the vitreous cavity (Fig. 5). We suspect that these differences in distribution speeds and patterns are attributable to certain pharmacodynamic properties of the drugs, such as differences in molecular weight and viscosity, which is a topic warranting further investigation.

In the present study, we carried out eye drop experiments using “enucleated” pig eyes that are inexpensive and readily available, because these were preliminary experiments and the selection of suitable solutions for human studies was also our goal. Following the administration of Gd and ^17^O, distribution in the anterior chamber was observed within 0.5 h, and we were able to visualize the distribution to the vitreous cavity at 1 h post-administration. In some previous studies, ^17^O distribution within the anterior chamber was detected after 4 min in sedated rabbits [6], and also after a few min in living human [7]. Moreover, although in both these studies, monitoring was continued for up to approximately 40 min after administration, no distribution was detected in the vitreous cavity. The findings from these in vivo studies are consistent with our ex vivo results. In the present study, however, we failed to observe any drainage of the administered solution from the vitreous cavity (images were obtained until 62 h; data not shown). This finding suggests that the temporal changes in intraocular water flow out may be considerably different from those in living humans.

Given their morphological and functional similarities to those of the human eyes, the eyes of pigs are often used as an ex vivo animal model in vision sciences and ocular pharmacology research [17–21]. It has been reported that the porcine sclera has almost the same permeability as that of the human sclera [22]. Moreover, the structure of the cornea, vitreous properties, and retinal morphology do not differ significantly from those of human eyes. In addition, the diameter of the cornea and the size of the eyeball are similar to those in humans. The major difference between these two mammals lies in the thickness of the cornea, which is approximately 1.7 times thicker in pigs (877 ± 14 μm) [22] than in humans (521 ± 32 μm) [23]. This may result in poorer drug permeability of the porcine cornea than in the human cornea. Accordingly, this can be considered a limitation of the present study. Consequently, as a next step, we will proceed to investigate intraocular distribution via the administration of Gd or ^17^O eye drops using high-sensitivity sequences in human studies.

## Conclusion

We demonstrated that visualization of distribution in the vitreous cavity via eye drops could be achieved with enucleated pig eyes using Gd and ^17^O but not with ophthalmic solutions. The use of UH-T2W may be a promising approach for the early and highly sensitive visualization of intraocular water penetration and distribution via eye drops.

## Data Availability

The data that support the findings of this study are not openly available due to reasons of sensitivity and are available from the corresponding author upon reasonable request.
